# Posterior-Only Approach for the Correction of Severe Post-tubercular Kyphosis

**DOI:** 10.7759/cureus.34685

**Published:** 2023-02-06

**Authors:** Adetunji Toluse, Taofeek Adeyemi, Solomon Samuel, Adebola Biala, Albert Izuka

**Affiliations:** 1 Orthopedic and Trauma Surgery, National Orthopaedic Hospital, Lagos, NGA

**Keywords:** pott’s disease-tuberculous spondylitis, kyphosis surgery, spine deformity surgery, costotransversectomy, posterior-only spine surgery, post-tubercular kyphosis, spinal tuberculosis

## Abstract

Tuberculosis of the vertebral column (Pott’s disease) accounts for up to one-half of musculoskeletal tuberculous infections. The eradication of the infective organism (*Mycobacterium tuberculosis*) is achievable with chemotherapy. However, such patients with spinal tuberculosis are at risk of developing spinal deformity, and 3%-5% of the patients develop severe deformity greater than 60°. A 30-year-old female presented with back pain of 11 years, discharging sinus, and progressively worsening kyphotic deformity of eight-year duration. She had completed a full course of anti-tubercular chemotherapy. Her neurological examination was within normal limits. Antero-posterior and lateral view radiographs showed osteolytic destruction and collapsed T12 and L1 vertebrae with a thoracic kyphosis of 90°. We did a single-stage posterior-approach closing-opening osteotomy surgery utilizing costotransversectomy (T12 and L1 corpectomy, the insertion of expandable titanium cage, T10 to L3 pedicle screw, and rod fusion). Postoperative kyphosis was 25°. Her motor and sensory functions remained preserved following surgery. The duration of follow-up was 18 months post operation. The mainstay of treatment of severe post-tubercular kyphosis (PTK) is surgery. The correction is complex and could be staged or with multiple approaches and consequent high risk of complications. A single-stage posterior-approach surgery is less invasive.

## Introduction

Tuberculosis of the vertebral column (also known as Pott’s disease) accounts for up to one-half of musculoskeletal tuberculous infections [1]. The eradication of the infective organism (*Mycobacterium tuberculosis*) is achievable with combination chemotherapy. However, such patients with spinal tuberculosis are at risk of developing spinal deformity, and 3%-5% of the patients develop severe deformity greater than 60° [2-4].

Persistent and/or progressive spinal deformity affects spinal biomechanics and is a cause of chronic back/neck pain. Mild deformity may be asymptomatic, thus requiring no treatment or correction. However, in moderate to severe deformity, patients present with long-term complications such as persistent spinal deformity and imbalance, chronic back/neck pain, cardiorespiratory symptoms, quadriparesis/quadriplegia, paraparesis/paraplegia with or without sphincteric dysfunction, and cosmetic concerns [5].

Various surgical procedures, which could be via anterior, posterior, or combined approaches, are available for the correction of the deformity. These surgical options entail debridement followed by anterior and/or posterior instrumentation with bone grafts/cages, pedicle screws, and rods with varying grades of spinal osteotomies depending on the severity of the deformity and desired correction [6].

We present the clinical and radiographic results of a patient with severe post-tubercular kyphotic deformity who underwent spinal deformity corrective surgery (closing-opening osteotomy) using a posterior-only approach.

## Case presentation

A 30-year-old female presented to the spine unit because of back pain that radiates to the thighs, worsening kyphotic deformity that was affecting her activities of daily living, and equally cosmetic concerns. She had prior presented to a peripheral hospital with back pain of 11 years, progressively worsening kyphotic deformity of eight years, and discharging sinus on the left lumbar region of six-year duration. There was associated weight loss and paraparesis. She was retroviral negative. They made a diagnosis of spinal tuberculosis with Gene Xpert (Cepheid, Sunnyvale, CA, USA). She completed a 12-month course of combination anti-tubercular chemotherapy (rifampicin, isoniazid, pyrazinamide, and ethambutol) with the resolution of paraparesis and discharging sinus prior to referral to our facility because of spinal deformity. She had no sphincteric dysfunction.

**Figure 1:**
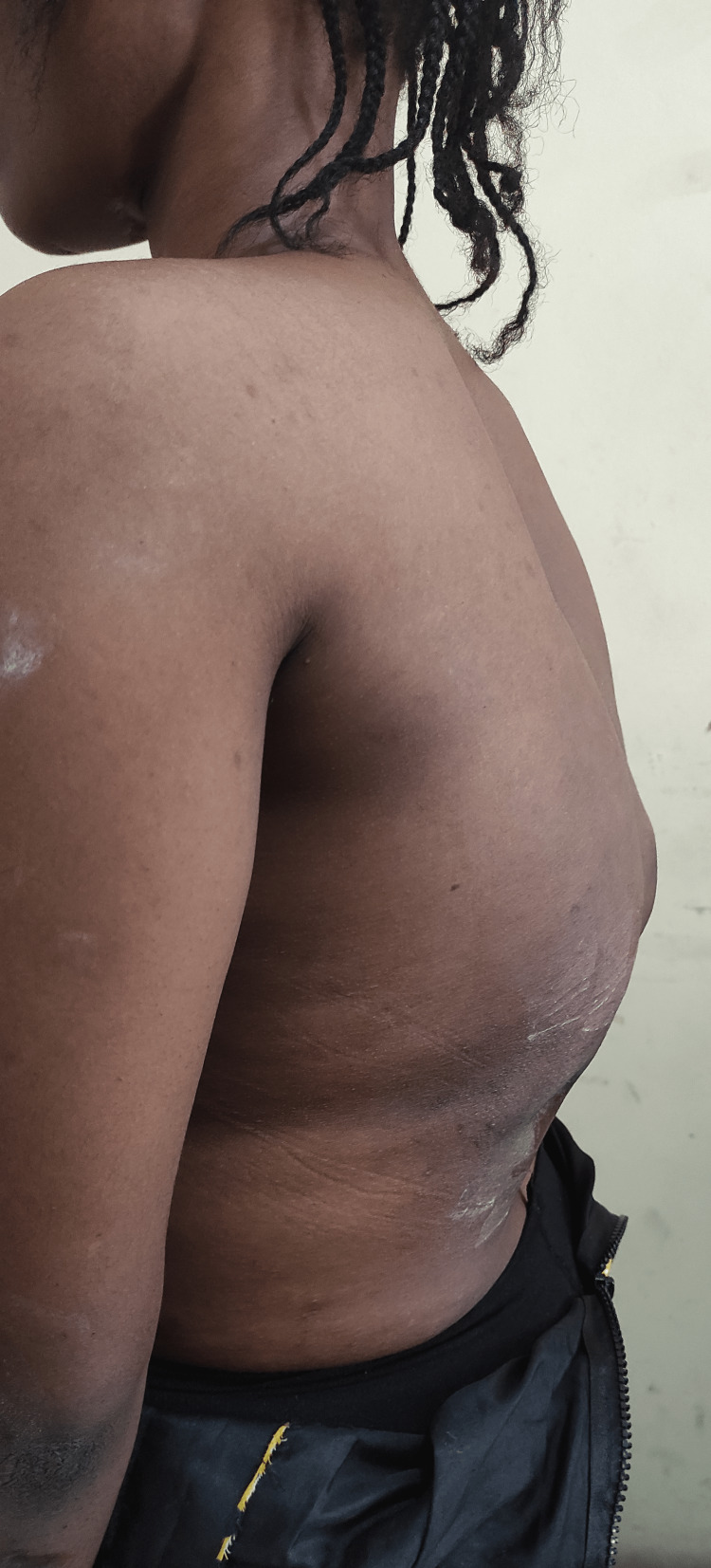
Clinical picture

**Figure 2:**
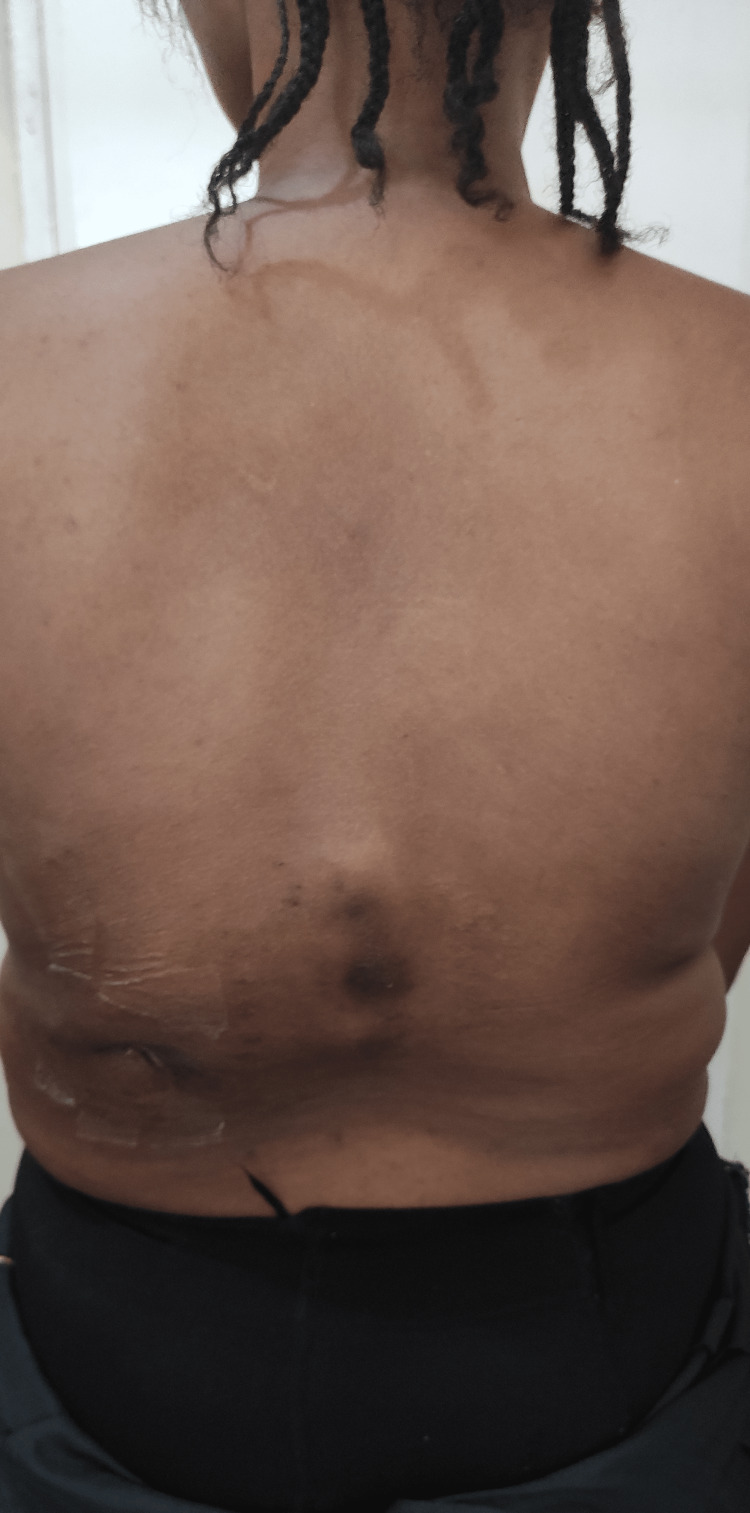
Clinical picture

Musculoskeletal examination revealed a young female with a thoracolumbar gibbus, a sinus scar, and hyperpigmented surrounding skin (Figures 1-2).

**Figure 3:**
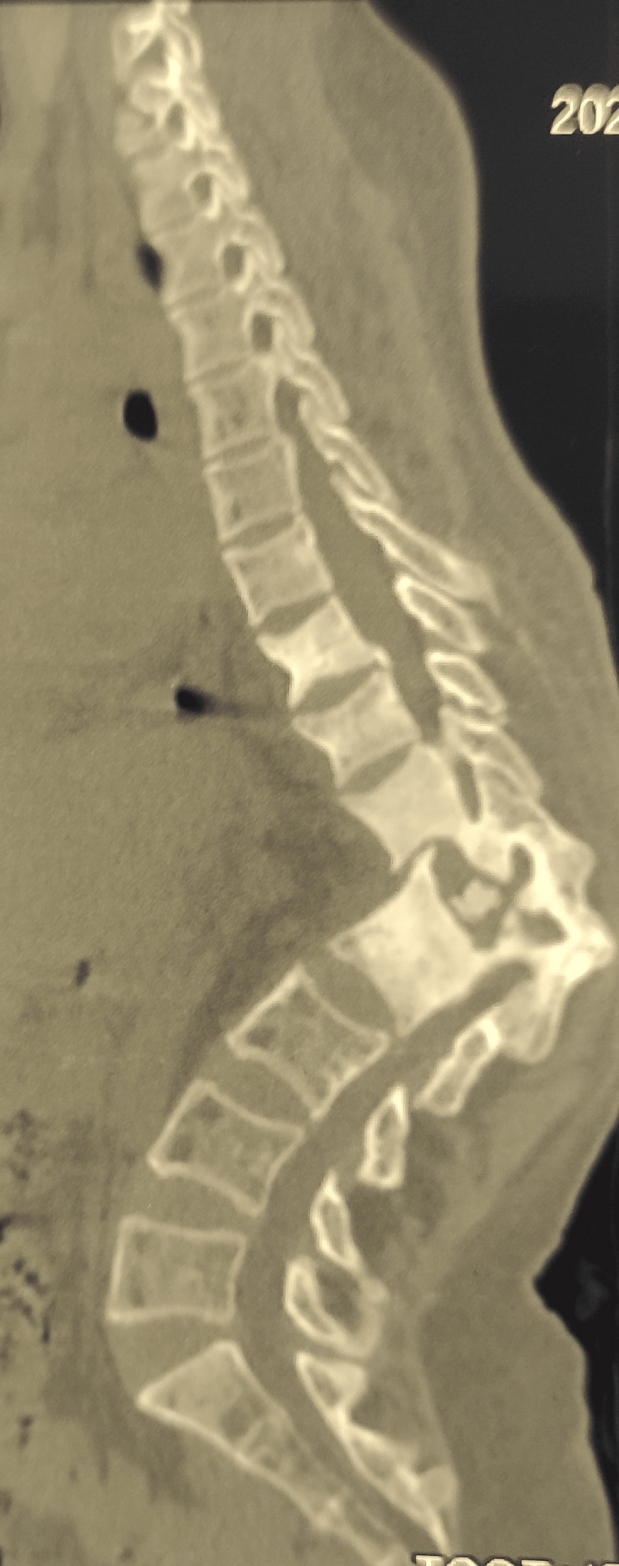
Computed tomography scan showing osteolysis and collapsed T12 and L1 vertebrae with severe kyphosis

**Figure 4:**
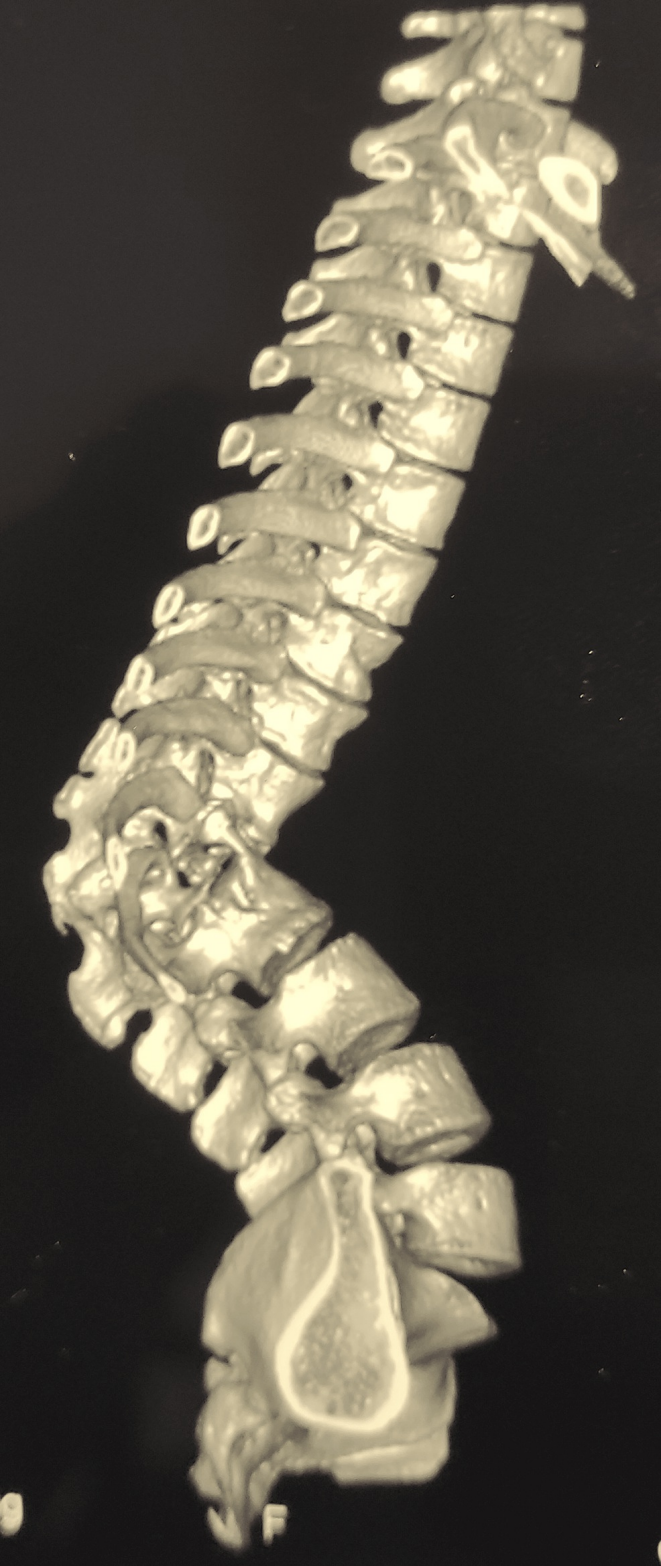
3D reconstruction of the spinal deformity 3D: three-dimensional

Motor power in both lower limbs was 5/5. Antero-posterior and lateral view radiographs showed osteolytic destruction and collapsed T12 and L1 vertebrae with kyphotic deformity of 90°. A three-dimensional reconstruction of the computed tomography scan showed further details of the severity of the pathology (Figures 3-4).

There was no associated scoliotic deformity. We made a diagnosis of severe post-tubercular kyphosis (PTK), and we worked her up for surgical correction via a posterior-only approach. We made a midline incision and after adequate dissection and exposure placed pedicle screws in T10, T11, L2, and L3 vertebrae. Thereafter, we performed T12 and L1 laminectomy, left T12 costotransversectomy to gain access to the anterior column of the spine for debridement, and corpectomy of the remnant of T12 and L1 vertebral bodies with curettes and pituitary rongeurs while taking adequate precaution to prevent pleura breach. We inserted the titanium expandable cage following the distraction of the anterior vertebral column. Deformity correction was by the combination of compression, distraction, and cantilever technique. We confirmed pedicle screws and cage position with intraoperative image intensifier. Following adequate hemostasis, we closed the wound in layers over a redivac drain. Bone biopsy carried out at surgery confirmed the diagnosis.

**Figure 5:**
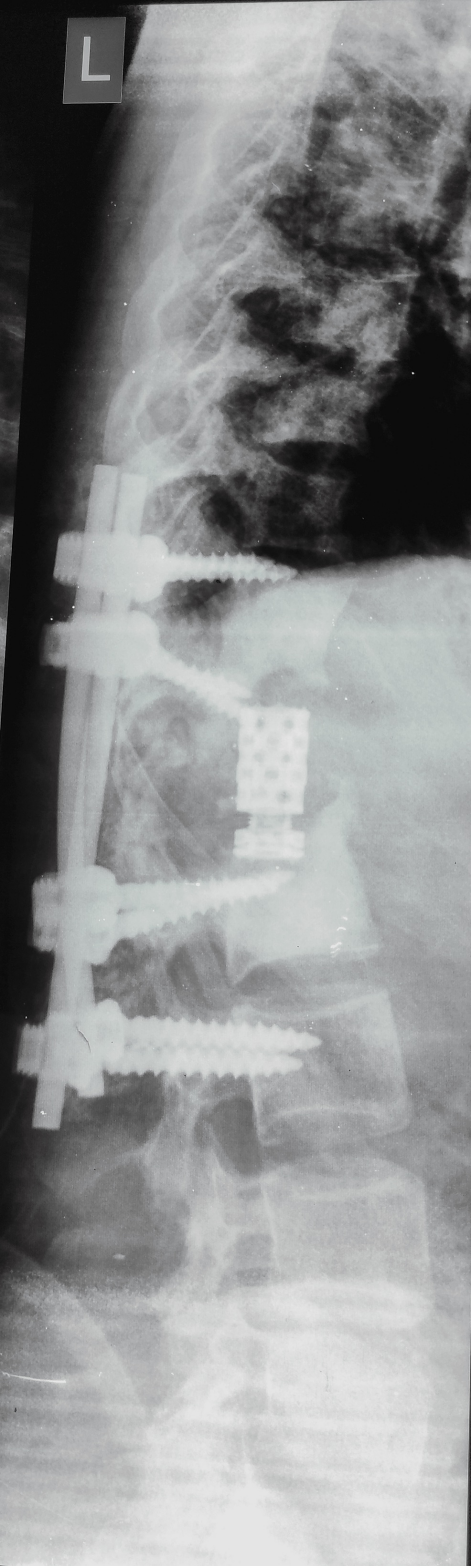
Postoperative radiograph

Immediate postoperative radiographs confirmed intact spine instrumentation with satisfactory deformity correction (Figure 5).

**Figure 6:**
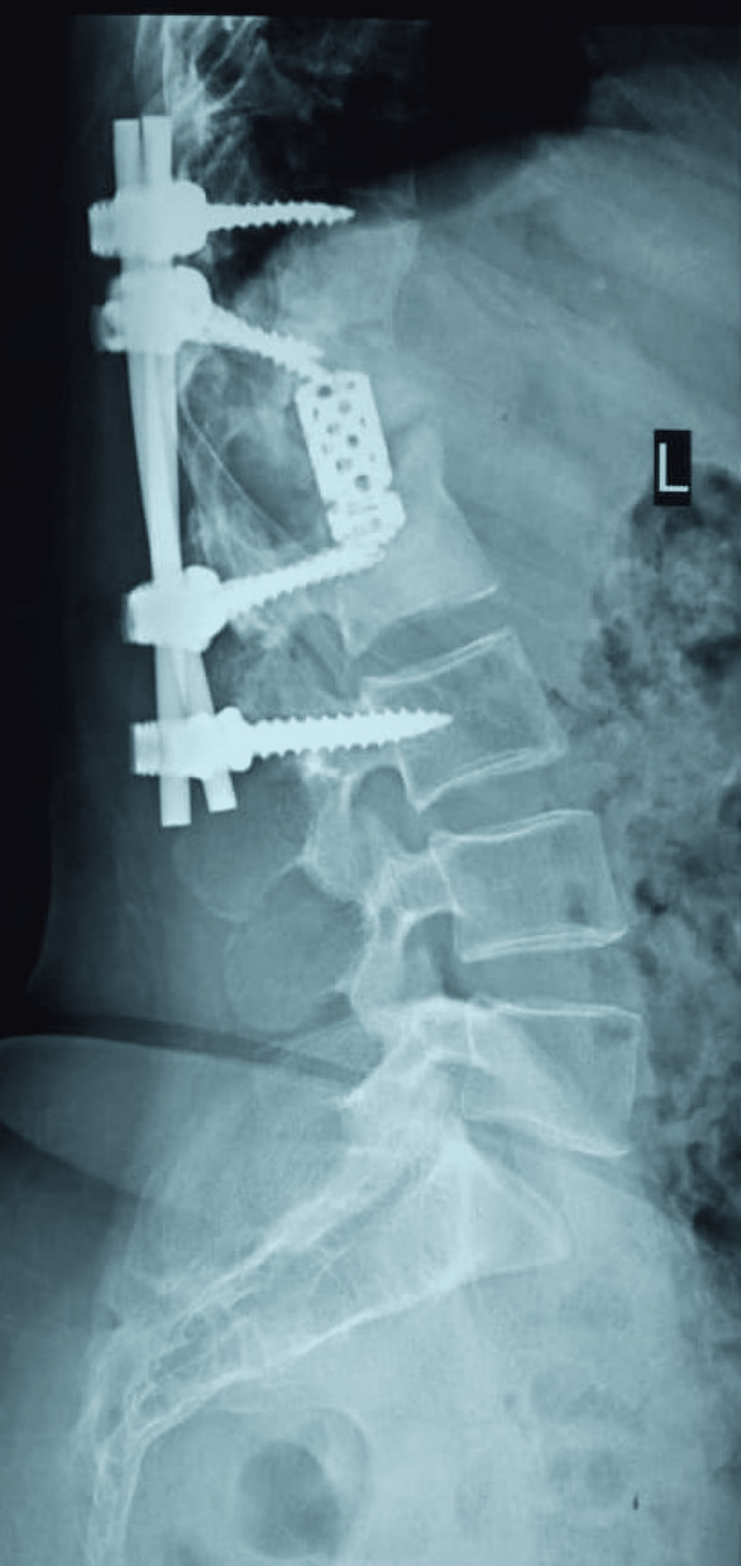
Eighteen months post operation

**Figure 7:**
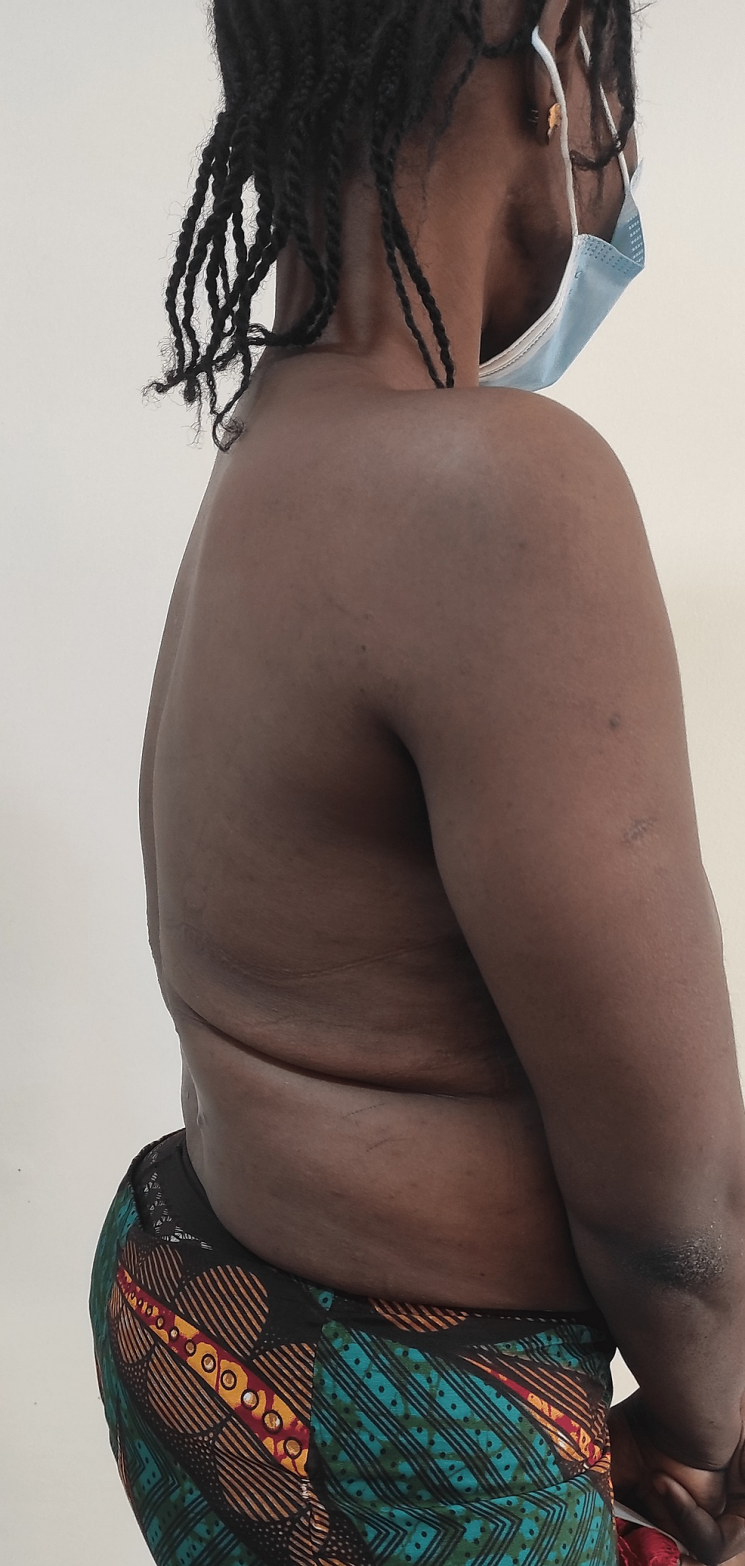
Post deformity correction

She had no neurological deficit. She began ambulation with support by the third day post operation and was discharged home walking without support at two weeks. Radiograph at 18 months post surgery revealed fusion with kyphotic angle of 25° (Figures 6-7).

## Discussion

Tuberculosis of the spine is a major underlying etiology of acquired kyphotic deformity of the spine in most parts of the globe [4]. Reports have shown that the spinal deformity that ensues from tuberculous spondylitis does persist and progress despite the completion of medical treatment [2-4]. The index patient presented with severe kyphosis after the completion of anti-tubercular therapy.

The indications for surgery in patients with spinal tuberculosis include failure of anti-tubercular chemotherapy with progressive osteolysis, severe pain, neurological impairment in the extremities with or without sphincteric dysfunction, worsening deformity with cosmetic concerns, and attendant low self-esteem [2,7].

The mainstay of treatment of severe post-tubercular kyphosis (PTK) is surgery. The correction is complex and could be staged or with multiple approaches and consequent high risk of complications. In severe kyphosis, several publications have highlighted the pitfalls of shortening the posterior column excessively and recommended simultaneous insertion of bone graft or metallic cage to open up and expand the hitherto collapsed anterior column (the closing-opening wedge osteotomy) [8-12].

Here, we have described a single-stage procedure and the good outcome of a closing-opening osteotomy for the correction of severe PTK, which shortens the posterior column and opens the anterior column appropriately with the insertion of an expandable cage to correct the deformity with the preservation of neurological function through a posterior-only approach.

## Conclusions

Despite advances in medicine and healthcare, we still have patients presenting with tuberculosis and/or its sequelae. Posterior-only approach with circumferential stabilization is effective in the correction of severe post-tubercular kyphotic deformity. Good outcomes are achievable with less morbidity compared to combination approaches.
